# A pilot, prospective trial of IntuBrite® versus Macintosh direct laryngoscopy for paramedic endotracheal intubation in out of hospital cardiac arrest

**DOI:** 10.1186/s12873-023-00845-3

**Published:** 2023-06-23

**Authors:** Przemysław Kluj, Michał Fedorczak, Tomasz Gaszyński, Paweł Ratajczyk

**Affiliations:** Department of Anesthesiology and Intensive Therapy, Medical University of Lodz, Lodz, Poland

## Abstract

**Background:**

Intubation in the case of out-of-hospital cardiac arrest (OHCA) is one of the most difficult procedures for Emergency Medical Services (EMS). The use of a laryngoscope with a dual light source is an interesting alternative to classic laryngoscopes. However, there are as yet no prospective data concerning the use of double light direct laryngoscopy (DL) by paramedics in traditional ground ambulance agencies in OHCA.

**Methods:**

We performed a non-blinded trial in a single EMS in Poland within ambulances crews, comparing time and first pass success (FPS) for endotracheal intubation (ETI) in DL using the IntuBrite® (INT) and Macintosh laryngoscope (MCL) during cardiopulmonary resuscitation (CPR). We collected both patient and provider demographic information along with intubation details. The time and success rates were compared using an intention-to-treat analysis.

**Results:**

Over a period of 40 months, a total of 86 intubations were performed using 42 INT and 44 MCL based on an intention-to-treat analysis. The FPS time of the ETI attempt (13.49 vs. 15.55 s) using an INT which was shorter than MCL was used (*p* < 0.05). First attempt success (34/42, 80.9% vs. 29/44, 64.4%) was comparable for INT and MCL with no statistical significance.

**Conclusions:**

We found a statistically significant difference in intubation attempt time when the INT laryngoscope was used. Intubation first attempt success rates with INT and MCL were comparable with no statistical significance during CPR performed by paramedics.

**Trial Registration:**

Trial was registered in Clinical Trials: NCT05607836 (10/28/2022)

## Background

In Europe, sudden out-of-hospital cardiac arrest (OHCA) is the third leading cause of death. Depending on how OHCA is defined, it affects more than 350,000 patients every year [1–4]. In European countries, with advanced Emergency Medical Services (EMS) systems, paramedics commonly perform endotracheal intubation (ETI) on patients with cardiac arrest to facilitate cardiopulmonary resuscitation (CPR) and protect the lungs from the aspiration of vomitus.

Intubation performed in direct laryngoscopy (DL) is one of several available options for an advanced airway management strategy during CPR. Alternatives to ETI include supraglottic airway (SGA) devices including the laryngeal mask airway (LMA), i-gel, and laryngeal tube. Compared with ETI, successful SGA insertion requires less training, it is rapid and simple, while at the same time offering ventilatory characteristics that are similar to ETI [5]. Despite controversy over the idealized form of airway management, multiple observational studies have reported better outcomes for patients with OHCA who received ETI via EMS. These patients are more likely to attain the return of spontaneous circulation (ROSC), survive until hospital admission, and survive neurologically intact when compared to patients upon whom SGA devices were used [6–12].

Intubation using a laryngoscope with a Miller or Macintosh blade is a difficult technique to master and requires fifty attempts to achieve a > 90% efficiency [13]. However, due to the appearance of modern laryngoscopes such as INT, it seems that less experienced operators can also perform successful intubation. According to our observation and specific comments provided by the paramedics, the real advantage of INT is associated with special embossing in the handle, that enables ergonomic grip of the laryngoscope handle and facilitates maneuvering during intubation. Additionally, paramedics emphasized that the angulated setting of the laryngoscope blade and the handle resulted in less use of strength and wrist movements to visualize the entrance to the larynx. On the other hand, every undertaken attempt required from opertaror more attention to not create leverage on the patient’s incisors.

Our objective was to assess and compare the time and effectiveness of ETI attempts at paramedic endotracheal intubation for nontraumatic adult OHCA. We also estimated the degree of difficulty of the intubation attempts.

## Methods

We received approval for this study from The Medical University of Lodz Ethics Board (Protocol Signature: RNN/06/20/KE). Trial was registered in Clinical Trials: NCT05607836. The study adheres to CONSORT guidelines.

### Study design

A multitude of observational evidence has suggested a possible survival advantage for tracheal intubation as compared with other airway management methods. However, instrumental airway management during OHCA still requires high-quality research on which to base treatment recommendations.

We designed a prospective clinical study to compare INT (IntuBrite®, LLC; Vista, CA, USA) and Macintosh classic laryngoscope (MCL) for ETI performed by paramedics in OHCA without an emergency physician on the scene. Our main goal was to determine whether INT is superior to using an MCL laryngoscope during a tracheal intubation attempt in nontraumatic OHCA, in terms of time and effectiveness.

**Fig. 1:**
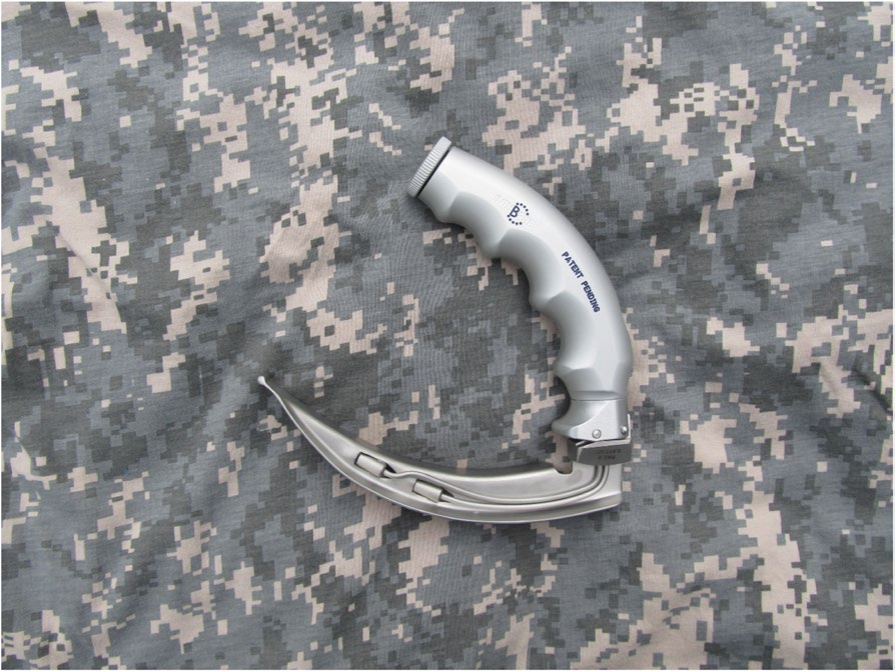
IntuBrite laryngoscope

The INT has changed the lighting method used – the commonly used white light is replaced by a system of two different light sources: ultraviolet light and light emitting diode type light. This type of lighting increases the image contrast and reduces glare on wet mucous membranes during DL. The laryngoscope handle is made from aluminium, thereby making it lightweight, and the ergonomic shape with finger embossing makes the laryngoscope comfortable and intuitive to use. The arched profile of the handle allows for improved manoeuvring during the intubation procedure which is important for less experienced operators (Fig. 1).

Paramedics enrolled in the study were recruited from among the large EMS ambulance services operating in Poland, which respond to emergencies for approximately 1.5 million people. All of the study participants were qualified to perform tracheal intubation in their current scope of practice. The paramedics were asked to secure the patients’ airways with intention to treat protocol utilizing 1 of the 2 laryngoscopes for the eligible patients in OHCA. No randomisation was performed, operators choose device based on their preference. No blinding was performed.

We conducted our study under actual field conditions over a period of 3 years to include a sufficient number of cases to perform valid statistical analysis. Our main goal was to assess the device within all 34 EMS crews but due to COVID-19 pandemic outbreak we stopped collecting data for the project. Therefore, we decided to analyze the data collected so far and title the study as a preliminary. All data gathered were claimed from nine ambulances crews.

As a final study design result eighty six patients were assessed. Ambulance crew was using INT for approximately 3–6 months. After that period, another EMS crew received INT for their use.

For this study, the sample size was based on G*Power 3.1 statistical tool, using a 2-tailed *t*-test. A minimum of 39 participants were necessary to achieve a Cohen d = 0.8 (alpha error = 0.05, and power = 0.95). To provide a safety margin in case of missing data, we increased the minimum size of the each study group to *n* = 42 and *n* = 44 patients.

### Setting and selection of patients

The trial population consisted of adults who had a nontraumatic OHCA. We included patients treated by paramedics employed in the Voivodeship Rescue Station (VRS) in Lodz City (Poland). VRS in Lodz responds to approximately 400,000 emergency calls per year. We examined prospectively collected data from the Medical Rescue Card generated by the Emergency Medical Support Command System (described in data collection section). Data were gathered from every eligible patient and stored in the VRS Lodz Data Warehouse.

We included all patients ≥ 18 years of age, treated by ground ambulances between 2016 and 2020, who experienced nontraumatic OHCA, as defined by all of the following: (A) absence of consciousness; (B) absence of palpable pulse; (C) absence of spontaneous breathing; and (D) Glasgow coma scale of 3; and initiation of chest compressions.

We excluded patients with confirmed or suspected traumatic cardiac arrest, those with missing data and the primary use of SGA by the paramedics. Patients with cardiac arrest occurring during hospital admission were excluded.

Following recommendations from The Medical University of Lodz Ethics Board (Protocol Signature: RNN/06/20/KE) the consent for publication was not obtained from patients nor next-of-kin of these participants.

### Interventions

The current standard of care in Poland is tracheal intubation using DL with MCL. The intervention consisted of an intubation attempt using the INT laryngoscope with a new type of lightning and an ergonomic handle shape.

A standard approach to airway management (from basic to advanced techniques) was agreed on by the participating paramedics. This included patient positioning, the use of bag-mask ventilation (BMV) and simple airway devices prior to advanced airway management. Video assisted laryngoscopy is not used by paramedics in Poland.

For tracheal intubation in OHCA, a 2-person technique using DL was recommended. During resuscitation in a two-person EMS team (current standard in Poland), one of the paramedics performs chest compressions over the patient’s head, while the other paramedic prepares all the necessary equipment to perform intubation, confirm the position of the tube and initiate ventilation. End-tidal carbon dioxide monitoring was used to confirm correct device placement in all patients. All study participants followed the European Resuscitation Council Guidelines for Resuscitation 2015 [14].

Paramedics were allowed to adjust the airway management technique during OHCA to specific patient and perceived needs. The trial protocol specified 2 attempts using each laryngoscope before SGA devices were used. It has to be emphasized that we only assessed first intubation attempt parameters. According to the terms of approval granted by the Ethics Board, paramedics were obliged to undertake every intervention that was in the patient’s best interest.

### Outcome and other measures

All outcomes were collected for all eligible patients and were reported by the paramedics using the Medical Rescue Card and trial protocol.

The primary outcome was the time of the first ETI attempt performed by the paramedics. The time was measured by paramedic itself using an electronic stopwatch in participant smartphone. Time measurement began when the paramedic held the selected laryngoscope and declared their readiness to perform the procedure. After intubation performance and putting away the laryngoscope, paramedic turned off the time measurement by pressing the touch screen of the phone with his finger. After performing the above-mentioned activities, operator proceeded to inflation of the tube sealing cuff and confirmation of its correct position by auscultation of the stomach, lungs and connecting the capnograph. Timing was paused before proceeding with the above-mentioned sequence.

The secondary outcome was the effectiveness of the first ETI attempt for instrumental airway management for nontraumatic adult OHCA. With the exception of standard clinical protocol, end-tidal carbon dioxide monitoring was used to confirm correct device placement.

As with other measures we collected the data concerning the degree difficulty of intubation attempts for all of the patients included in the study. Instead of Cormack-Lehane classification, a short questionnaire consisting of four options to select (easy, complicated, difficult and very difficult) was used to examine participants’ subjective experiences of different intubation techniques. A long-term survival and a cost-effectiveness analysis were not included within this trial.

### Data collection and analysis

We obtained all of the relevant data from the Voivodeship Rescue Station in Lodz Data Warehouse. Outcome data were collected from the Medical Rescue Card and from trial protocol delivered by participating paramedics. The estimated time was counted to two decimal places. Efficacy was determined using the zero-one system. We abstracted basic demographic data including the sex and age of all of the patients.

All statistical analyses were performed using PQStat (Version 1.6.8.304.) and IBM SPSS (Version 24.0). We present the data as mean values, with standard deviation (SD), or medians, with interquartile range (IQR), where appropriate. The time (seconds) of the first ETI attempt, depending on the laryngoscope used, was compared using a Student’s *t*-test (parametric values) after verifying the normality of the distribution with the Kolmogorov-Smirnov test and the homogeneity of variance with the Fisher-Snedecor F-test. The effectiveness and the degree of difficulty of the ETI attempts depending on the laryngoscope used were compared using the chi-square test. We present adjusted odds ratios (aOR) with 95% confidence intervals.

A *P* value of ≤ 0.05 was considered statistically significant and a *P* value of < 0.01 was considered to be highly significant.

### Data safety and monitoring

Data quality assessments are executed routinely by utilizing the Emergency Medical Support Command System, which is part of the National EMS Command System. Quality-assurance initiatives are conducted regularly to ensure completeness and accuracy. After every ETI attempt during CPR by paramedics, the research team sent a self-report questionnaire to the paramedic team to acquire specific data.

## Results

We collected 93 case report forms (CRFs) from patients after OHCA within a 40-month period. For various reasons 7 CRFs were excluded from further evaluation: (3) reports were missing case data, (1) was collected on admission to the hospital, (3) primary use of LMA. Finally, the CRFs of *n* = 86 patients were included, *n* = 42 with the use of INT and *n* = 44 with MCL.

**Fig. 2:**
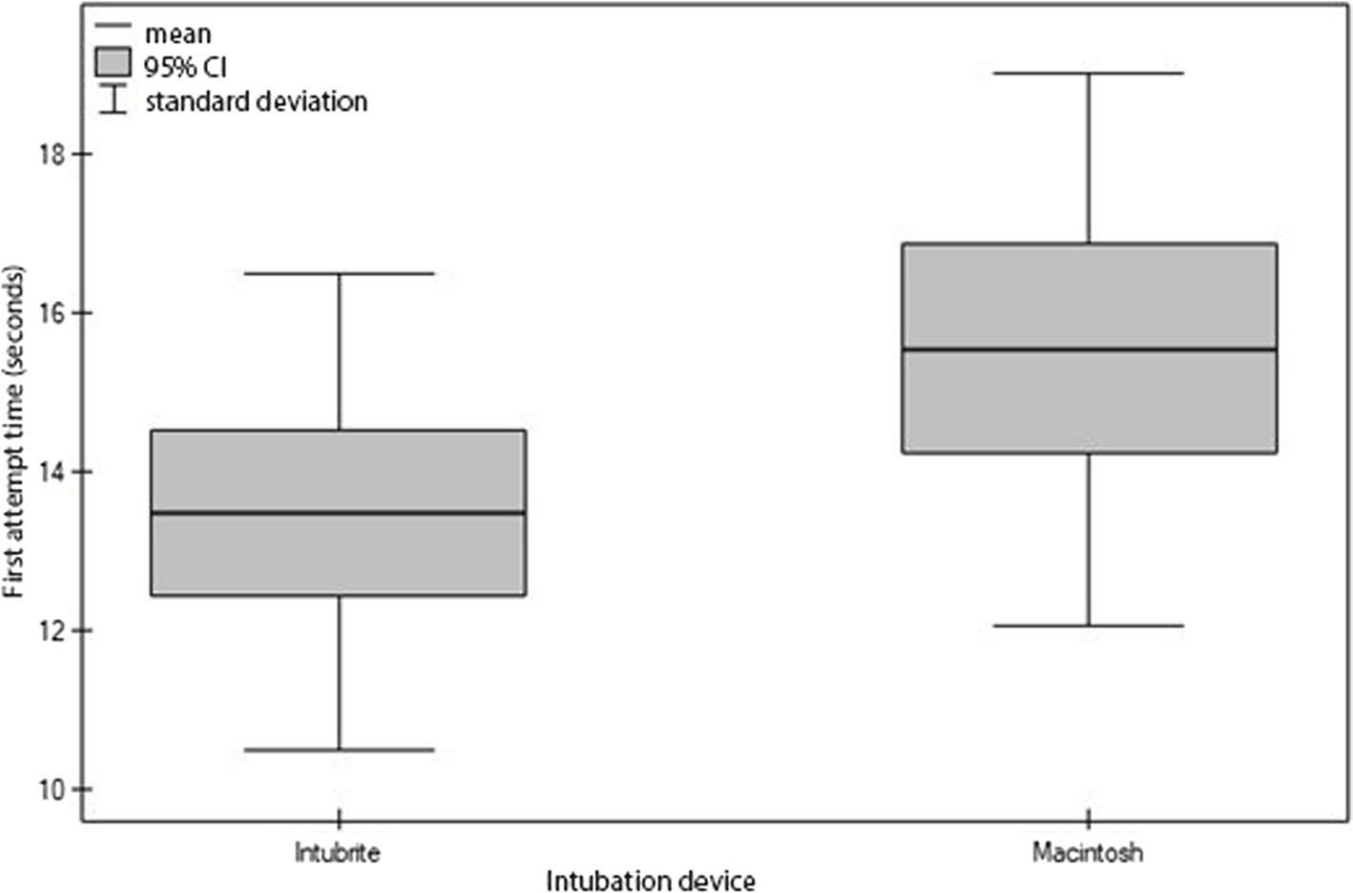
First attempt time (seconds) for each device

The average professional experience of the paramedics in instrumental airway management was 6 years (DL and LMA). The primary outcome was the FPS time of the ETI attempt performed by the paramedics. The mean time of intubation using INT (13.49) is shorter than when MCL was used (15.55). The difference of means is 2.05 and is statistically significant (*p* < 0.05) (Fig. 2; Table 1).

**Table 1:** First attempt time (seconds) for each device

	Intubation device	
	IntuBrite	Macintosh
Arithmetic mean	13.49	15.55
Standard deviation	3.00	3.48
Minimum	9.86	9.87
Lower quartile	11.37	12.84
Median	12.92	15.84
Upper quartile	14.50	17.85
Maximum	22.25	23.16
Kolmogorov-Smirnov test	D = 0.1607, df = 34, *p* = 0.3096	D = 0.1060, df = 29, *p* = 0.8670
Fisher-Snedecor F-test	*F* = 0.7429, *p* = 0.4106	
Student’s t-test	t = 2.5190, df = 61, *p* = 0.0144	

There was a significant (*p* < 0.05) difference in mean first attempt times (seconds) depending on the device used. In the case of the IntuBrite, the mean time is lower than when the Macintosh was used. The difference in means is 2.0577.

The secondary outcome was the success rate on the first intubation attempt. First attempt success (34/42, 80.9% vs. 29/44, 64.4%) was comparable for INT and MCL. The difference found was not significant (*p* = 0.08) (Table 2).

**Table 2:** Effectiveness for each device

Effectiveness	Intubation device			
	IntuBrite		Macintosh	
	Number	Percentage	Number	Percentage
unsuccessful	8	19.05%	16	35.56%
successful	34	80.95%	29	64.44%
Chi-square test of independence	chi^2 = 2.9636, df = 1, *p* = 0.0852			

There was no significant (*p* > 0.05) relationship between the effectiveness and the type of device used.

As with the other measures, we evaluated the degree of difficulty of intubation attempts for all of the patients included in the study. The collected data did not differ significantly between the INT group and the MCL group (*p* = 0.67) (Table 3).

**Table 3:** Degree of difficulty for each device

Degree of difficulty	Intubation device			
	IntuBrite		Macintosh	
	Number	Percentage	Number	Percentage
Very difficult	5	11.9%	7	15.91%
Difficult	5	11.9%	9	20.45%
Complicated	5	11.9%	8	18.18%
Easy	27	64.29%	20	45.45%
Chi^2 test of independence	chi^2 = 3.1663, df = 3, p = 0.3667			

There was no significant (*p* > 0.05) relationship between the degree of difficulty and the type of device used.

### Limitations

The first limitation of our study was the time period. The use of intubation is a rare event for paramedics in Poland, therefore, a period of over 3 years was needed to investigate a sufficient number of cases. The enrolled paramedics were from a large, but single EMS area. For that reason, our study sample was small and unbalanced. The obtained results should not be generalized.

A second important limitation of our study is the lack of availability of videolaryngoscopes in the Polish EMS under consideration. The current standard of care in Poland in instrumental airway management is SGA or tracheal intubation using DL. Obtaining a comparison between videolaryngoscopy and standard DL in OHCA performed by other authors [15, 16] would require time under Polish conditions. For these reasons, a comparison between the classic laryngoscopes and the new type of laryngoscopes requires further investigation.

A third limitation of the study was a self-report formula used for data evaluation obtained from participants after every ETI attempt performed during CPR. The use of a professional method of evaluation would make the work more attractive.

## Discussion

Airway management is a crucial procedure in the management of OHCA. ETI has long been considered as the criterion standard for securing the airway in prehospital care. The experience of paramedics concerning intubation is limited and has been questioned as the preferred method of advanced airway management [17, 18]. DL might have improved the time and success rates using new types of classic laryngoscopes among occasional operators. The recent focus on airway management utilizing videolaryngoscopy is very important and interesting but cannot be transferred into every EMS system in Europe [15, 16].

The objective of our study was to estimate the between-group difference in the time and effectiveness of first ETI for adult patients with OHCA treated by paramedics utilizing either an MCL or INT laryngoscope for tracheal intubation as their initial advanced airway management strategy. We also estimated the degree of difficulty of intubation attempts.

Results obtained from this prospective out-of-hospital clinical trial demonstrate the improved time of FPS of ETI during CPR with an INT laryngoscope. This is a very interesting finding considering the lack of previous clinical experience of Polish paramedics in using this type of laryngoscope. As yet, there is no data in the literature comparing the use of INT in OHCA with other devices. Few previous studies showed a significantly longer FPS time during mannequin CPR compared to our study [19, 20]. However, the results obtained from a simulation cannot simply be transferred to the out-of-hospital setting. According to our study, improved FPS time for INT may be due to two light sources and the facilitated manoeuvring option, which is important for less experienced operators.

A failed initial intubation attempt was shown to be associated with an average delay of 3 min in the time to ROSC within the first 15 min of CPR [21]. However, due to the appearance of modern laryngoscopes, the success rate concerning the first intubation attempt between INT and MCL were similar and the difference found in our study was not significant. Results for DL intubation effectiveness obtained in our study are similar to those obtained from simulation studies with mannequins [19, 20] and real patients [5, 15, 16]. In our study, we were not able to show an increased success rate for an ETI attempt when performed by Polish paramedics. Despite the extensive use of videolaryngoscopes in most of the available studies, it was found that the effectiveness of FPS intubation compared to DL is similar [15, 16, 22, 23]. Collected data according to the degree of difficulty of intubation attempts did not differ significantly between the INT group and the MCL group.

For subjects with OHCA, emergency intubation carries a higher risk of complications than elective airway management in the operating room or ICU. Time spent on successful ETI in prehospital care has to be reduced in order to allow for the application of an improved focus on the provision of optimal CPR, which confers a survival benefit for OHCA [24]. For these reasons, our study results should not be generalized, and further investigation is needed.

## Conclusions

In our study ETI attempt time using INT was shorter than when MCL was used. The difference found was statistically significant. We found no difference in FPS between INT and MCL laryngoscopes during CPR by paramedics.

## Data Availability

Please contact author for data requests.

## Abbreviations

OHCA: Out-of-hospital cardiac arrest
ETI: Endotracheal intubation
ROSC: Return of spontaneous circulation
EMS: Emergency medical service
DL: Direct laryngoscopy
CPR: Cardiopulmonary resuscitation
SGA: Supraglottic airway devices
VRS: Voivodeship Rescue Station
MCL: Macintosh laryngoscope
INT: IntuBrite
FPS: First pass success
BMV: Bag-mask ventilation
CRF: Clinical report form
